# Can probiotics be an alternative to chlorhexidine for oral care in the mechanically ventilated patient? A multicentre, prospective, randomised controlled open trial

**DOI:** 10.1186/s13054-018-2209-4

**Published:** 2018-10-28

**Authors:** Bengt Klarin, Anne Adolfsson, Anders Torstensson, Anders Larsson

**Affiliations:** 1 Department of Anaesthesiology and Intensive Care, Lund University and Skåne University Hospital, SE-221 85 Lund, Sweden; 2 Department of Anaesthesiology, County Hospital, Halmstad, Sweden; 3 Department of Surgical Sciences, Section of Anaesthesiology and Intensive Care, Uppsala University Hospital, Uppsala, Sweden

**Keywords:** Oral care, Mechanically ventilated patients, Probiotics, *Lactobacillus plantarum* 299, Chlorhexidine, Resistance to antibiotics, Ventilator-associated pneumonia

## Abstract

**Background:**

Pathogenic enteric bacteria aspirated from the oropharynx are the main cause of ventilator-associated pneumonia (VAP). Using chlorhexidine (CHX) orally or selective decontamination has been shown to reduce VAP. In a pilot study we found that oral care with the probiotic bacterium *Lactobacillus plantarum* 299 (Lp299) was as effective as CHX in reducing enteric bacteria in the oropharynx. To confirm those results, in this expanded study with an identical protocol we increased the number of patients and participating centres.

**Methods:**

One hundred and fifty critically ill patients on mechanical ventilation were randomised to oral care with either standard 0.1% CHX solution (control group) or a procedure comprising final application of an emulsion of Lp299. Samples for microbiological analyses were taken from the oropharynx and trachea at inclusion and subsequently at defined intervals.

Student’s *t* test was used for comparisons of parameters recorded daily and Fisher’s exact test was used to compare the results of microbiological cultures.

**Results:**

Potentially pathogenic enteric bacteria not present at inclusion were identified in oropharyngeal samples from 29 patients in the CHX group and in 31 samples in the probiotic group. Considering cultures of tracheal secretions, enteric bacteria were found in 17 and 19 samples, respectively. Risk ratios show a difference in favour of the Lp group for fungi in oropharyngeal cultures. VAP was diagnosed in seven patients in the Lp group and in 10 patients among the controls.

**Conclusions:**

In this multicentre study, we could not demonstrate any difference between Lp299 and CHX used in oral care procedures regarding their impact on colonisation with emerging potentially pathogenic enteric bacteria in the oropharynx and trachea.

**Trial registration:**

ClinicalTrials.gov, NCT01105819. Registered on 9 April 2010.

First part: Current Controlled Trials, ISRCTN00472141. Registered on 22 November 2007 (published *Critical Care* 2008, 12:R136).

## Background

Ventilator-associated pneumonia (VAP) occurs in 5–50% of mechanically ventilated patients in the intensive care unit (ICU) and leads to increased costs and length of stay in ICUs and hospitals [1–5]. VAP develops primarily due to aspiration of microorganisms in either oropharyngeal secretions or fragments of biofilm from the endotracheal tube (ETT) [6]. Also, clearance of secretions is compromised by sedating medication that is commonly used during mechanical ventilation. Approaches applied to address VAP are proper implementation of prevention bundles [7, 8] or use of ETTs with a subglottic suction lumen. However, although these methods may reduce the risk of aspiration, they do not lower the prevalence of oral pathogens. A reduction in those organisms can be accomplished with antibiotics used in strategies such as selective oropharyngeal decontamination (SOD) and selective digestive decontamination (SDD) that have been found to decrease the incidence of VAP and improve survival [9–13].

Unfortunately both of these methods are also associated with a non-negligible risk of development of resistant bacteria [14–17]. Chlorhexidine (CHX) is an antiseptic used for oral care in intubated patients in many ICUs and has been demonstrated to reduce the incidence of VAP [18–20]. On the other hand, similar to antibiotics, CHX induces bacterial resistance and hypersensitivity [21–23].

Health care-related infections are an increasing problem. In times of increasing problems of antibiotic-resistant bacteria there is a need for sustainable solutions in the struggle against health care-related infections. By using competing stable probiotics the need for antibiotics and antiseptics, which both have properties that may increase the development of new resistant strains of pathogens, can be reduced. Therefore, we investigated whether the probiotic bacterium *Lactobacillus plantarum* 299 (Lp299) (DSM 6595), which by its ability to adhere to the mucosa via a mannose-specific mechanism to the mucosa [24] throughout the gastrointestinal tract represents a competing microbiological mechanism by blocking sites for possible pathogenic microorganisms, could serve as an alternative to CHX or other procedures for oral care in mechanically ventilated ICU patients. The genomically closely related Lp299v is a common probiotic additive in various juices (marketed as ProViva®) and milk products that have been widely sold for more than 25 years without any known side effects. Both of these strains adhere to the mucosa throughout the gastrointestinal (GI) tract [25–27] to the same extent in antibiotic-treated intensive care patients as in healthy volunteers and thus reinforce the ability to withstand colonisation of potential pathogens. In a single-centre pilot study in 50 patients [28] we found that the Lp299 was more effective than CHX in reducing colonisation with enteric pathogens. Therefore, we extended that initial evaluation by including another 100 patients and two additional centres. Thus, a total of 150 patients were enrolled under an identical protocol with the aim of ascertaining whether the treatment results would be consistent, and here we report the data obtained in the expanded study.

## Methods

The study was approved by the local Human Ethics Committee/Review board and registered at Current Controlled Trials (ISRCTN00472141) and ClinicalTrials.gov (NCT01105819). Informed consent was obtained from the patients or their next of kin.

The initial part of the study (50 patients, included 29 January 2004–22 March 2007) was performed in the ICU of the Department of Anaesthesiology and Intensive Care, Skåne University Hospital, Lund, Sweden, and the extended part (100 additional patients, included 27 May 2010–26 January 2015) in the same facility as well as in the ICUs at Central Hospital, Kristianstad and County Hospital, Halmstad, both also in Sweden. The patients were followed for 6 months from inclusion regarding hospitalisation and mortality.

The identical protocol and CRF were used in both parts of the presented study. There were no changes in the routine care regarding VAP prophylaxis. The patients were randomised via sealed envelopes into groups of 10 at a 1:1 ratio to receive either a standard oral treatment (control group) or the study treatment with Lp299 (Lp group). The day of inclusion was designated day 1. To be included in the study, patients had to fulfil the following criteria: 18 years of age or older; critically ill with an anticipated need for mechanical ventilation of at least 24 h; not moribund; not having pneumonia as admission diagnosis; no fractures in the facial skeleton or the base of the skull; no oral ulcers; not immune deficient; not a carrier of HIV or viral hepatitis; not being tracheotomised; endotracheal intubation and mechanical ventilation initiated within 24 h before inclusion; and no standard oral care had been performed.

After screening, the patients were included when they showed circulatory and ventilatory stability and before the first oral care procedure. The oral care procedure was performed twice a day. The control group was treated according to the department’s standard protocol: dental prostheses were removed; secretions were removed by suction; teeth were brushed using toothpaste (Zendium; Opus Health Care, Malmö, Sweden); and all mucosal surfaces were cleansed with swabs that had been moistened with a 1 mg/ml CHX solution (Hexident; Ipex, Solna, Sweden). In the Lp group the initial mechanical steps were the same as in the control group, but the subsequent cleansing was instead performed with gauze swabs soaked in carbonated bottled water, after which Lp299 was applied to the mucosal surface of the oral cavity. This application was done using two gauze swabs (one for each side of the oral cavity) that had been allowed to absorb 10 ml of a solution containing a total of 10^10^ colony-forming units (CFU) of Lp299; excess suspension was not removed. In both groups, when necessary between the oral care procedures, secretions were removed by suctioning and gauze swabs moistened with carbonated bottled water were used to wipe off remaining secretions. Participation in the study was ended after tracheal extubation or when the patient was discharged from the ICU.

Cultures were taken from the oropharynx and from the trachea at inclusion. Sampling was repeated before the oral care procedures on days 2, 3, 5, 7, 10, 14 and 21 in patients who were still mechanically ventilated. If the tracheal extubation occurred on a non-culture day, cultures were taken before the extubation. One set of cultures was analysed according to normal routines at the Department of Clinical Microbiology, Skåne University Hospital, Lund and at the Department of Clinical Microbiology, County Hospital, Halmstad for the patients at the respective facilities. Another set was sent blinded to the research laboratory at Probi AB, Lund, Sweden for identification and quantification of total CFU of lactobacilli and identification of Lp299.

In the first part of the study (50 patients) [28] viable counts of lactobacilli were obtained on Rogosa agar (Oxoid, Basingstoke, UK) incubated anaerobically at 37 °C for 3 days. Colonies suspected to be Lp299 (large, creamy white–yellowish and somewhat irregular in shape) were selected and identified by randomly amplified polymorphic DNA typing [29]. In the extended part of the study Lp299 was identified with a PCR technique. In short, DNA for analysis was extracted in an Arrow instrument (DiaSorin INUK, Dublin, Ireland) and eluated DNA was used in an absolute quantitative real-time PCR assay for *Lactobacillus plantarum*. After some optimisation of the concentrations of primers and probe (Applied Biosystems, Woolston, UK) the assay was performed on a Lightcycler 96® instrument (Roche Diagnostic GmBH, Mannheim, Germany) with a Fast Start Essential DNA Probe Master® (Roche Diagnostic GmBH) [30]. DNA for the standard curve was extracted from overnight cultures of *L. plantarum* 299 in MRS, incubated at 37 °C. The qPCR programme was as follows: 10 min at 95 °C followed by two-step amplification at 95 °C for 10 s and 57 °C for 10 s for 45 cycles.

In the initial part of our study [28] we found that the Lp299 became established in the oropharynx in all treated patients. Therefore, in the extended investigation we analysed Lp299 only in the tracheal secretion samples.

In both study parts we registered the ICU admission diagnosis according to the ICD-10, APACHE II and daily SOFA scores, and in the extended part of the study also the Simplified Physiology Score 3 (SAPS 3). Respiratory parameters, including the Lung Injury Score (LIS), blood gas analysis, and CRP and white blood cell (WBC) counts, were registered daily throughout the investigations, as was information on parenteral/enteral administration of drugs and fluids, nutrition and total volumes, and vomiting and gastric residual volumes.

Enteral nutrition was started when the patients were circulatory and respiratory stable, and was subsequently increased according to the department’s protocols. Stress ulcer prophylaxis (esomeprazole iv; Astra Zeneca, Södertälje, Sweden) was given from admission until enteral nutrition was fully established (i.e. for 3–4 days for most patients). Prescribed antibiotics and corticosteroids were also registered.

As indicated in Statistics, the study was not powered to detect any differences in the incidence of VAP. However, VAP was recorded and identified using the following criteria: a new, persistent or progressive infiltrate on chest radiograph combined with at least three of the other four criteria; a purulent tracheal aspirate; positive culture of tracheal aspirates occurring after 48 h of mechanical ventilation; rectal or urine bladder temperature higher than 38.0 °C or less than 35.5 °C; and WBC count more than 12 or less than 3 [31], or a rapid increase in WBC count without suspicion of infection in another organ.

For the main duration of the mechanical ventilation period, the patients were placed in a semi-recumbent position and ventilated in a pressure-controlled or pressure-supported mode. A heat moisture exchange filter was used routinely, but active humidification was applied if secretions were more viscous. Suctioning of the airways was performed with a closed suction system (TRACH-Care 72; Ballard Medical Products, Draper, UT, USA). Most of the patients inhaled 2.5 mg of salbutamol (GlaxoSmithKline, Solna, Sweden) and 0.5 mg of ipratropium (Boehringer Ingelheim, Stockholm, Sweden) every 6 h.

### Statistics

As mentioned in the Introduction the number of patients assessed in this expanded investigation was chosen with the intension of confirming the results obtained in the initial study. A primary power calculation was made addressing the VAP issue. Anticipating a halving from 12% of the VAP incidence for the probiotic group compared to the standard procedure group, a total number of 778 patients would have been required. With this knowledge we decided to make this first expansion aiming at a confirmation of our primary results.

Statistical methods were selected after consulting a biostatistician, and the statistical analyses were performed using Microsoft Excel 2011 (Microsoft, USA) and SAS version 9.4 (SAS, USA). All analyses were carried out on the total number of participants (50 + 100). The study was not powered to detect any difference in VAP incidence.

Student’s *t* test was used for comparisons of the daily observations. Fisher’s exact test was employed to compare the results of microbiological cultures. *p* < 0.05 was considered significant. Risk ratios were calculated between the probiotic and control groups for the different groups of microbes.

## Results

After screening, a total of 150 patients (50 from the initial cohort + 100 in the extended investigation) were included in the present analysis, and 69 patients (23 initial cohort + 46 extended investigation) in the Lp group and 68 patients (21 initial cohort + 47 extended investigation) in the control group completed the study. Thirteen patients were excluded because they had < 24 h of mechanical ventilation or consent was withdrawn. Five and eight Lp and control patients respectively were tracheotomised. One patient in the control group was transferred to another hospital, but all of the others completed their study participation with the respective treatment.

We found no significant differences between the Lp group and the control group with respect to demographic data, number of ventilator days, length of stay (LOS) or ICU and in-hospital mortality (Table 1). Furthermore, we observed no major disparities regarding laboratory test results, severity scores or drug treatments. Cephalosporins, imipenem/cilastatin and piperacillin/tazobactam were the most frequently used antibiotics.

**Table 1:** Patient characteristics and admission diagnosis

	Lp299 group	Control group
Characteristic		
Age	66 (57–76)	65.5 (53.75–75)
Sex, male/female	40/29	36/32
APACHE II score	22 (18–27)	24 (18.75–28)
SAPS 3^a^	64.5 (54–75)	64.0 (54.5–76.5)
ICU mortality (*n*)	10	11
Additional in-hospital mortality (*n*)	14	12
ICU stay (days)	7.67 (3.58–11.54)	6.59 (3.24–9.82)
Ventilator days (invasive)	4.79 (0.38–24.52)	4.23 (0.75–22)
Diagnosis at ICU admission (*n*)		
Sepsis, bacteraemia, septic shock, meningitis	19	15
Cardiac arrest and cardiac failure	20	20
Respiratory insufficiency	6	11
Abdominal condition	8	9
Vascular condition	2	4
Trauma	5	2
Other	9	7

Data presented as median (first–third quartiles) except for sex, death rates and diagnoses. Differences are not significant

*APACHE* Acute Pathophysiology and Chronic Health Evaluation, *ICU* intensive care unit, *Lp299 Lactobacillus plantarum* 299, *SAPS* Simplified Acute Physiology Score

^a^Only for the later 100 patients

### Microbiological results

**Fig. 1:**
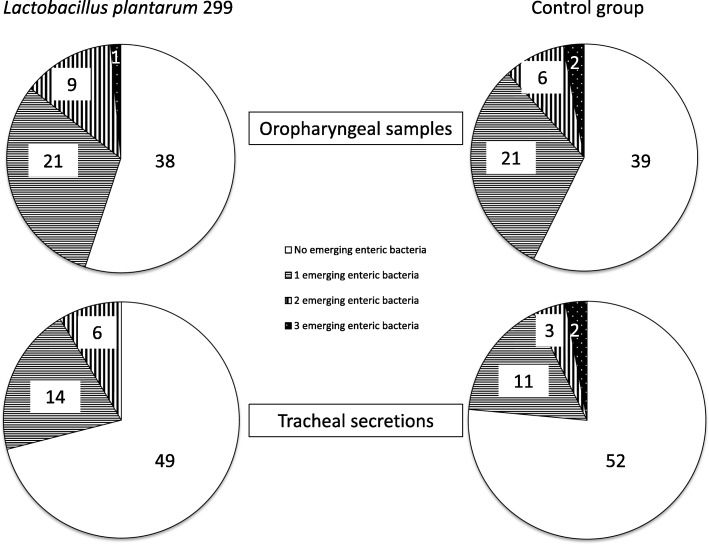
Results of oropharyngeal and tracheal secretion cultures. Number of patients presented for treatment group and culture type respectively. Presented figures are number of patients with and without new emerging enteric bacteria, not identified at inclusion

**Fig. 2:**
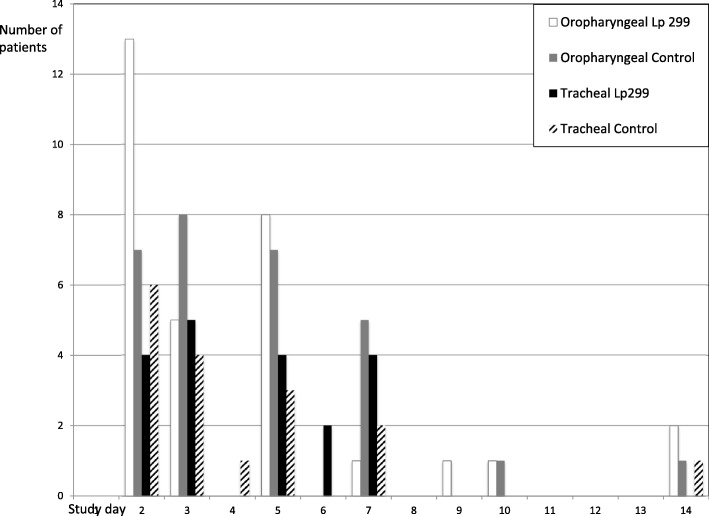
Distribution of the findings of emerging enteric bacteria. Pattern of identified emerging enteric species is somewhat scattered. On day 2, number of affected patients higher in Lp group but on day 3 results reversed. This may indicate slower action of probiotic bacteria compared to chemical effect of chlorhexidine. Gradual decrease of patients remaining in study similar in both groups. Lp299 *Lactobacillus plantarum* 299

The total number of new emerging bacteria or fungi did not differ significantly between the two groups. Thus, the numbers of patients with new enteric bacteria in the Lp299 and CHX groups were similar (Table 2); 31 and 29 for oropharyngeal cultures, and 19 and 17 for tracheal cultures, respectively. The distribution of patients without and with one, two and three new emerging enteric bacteria respectively are shown in Fig. 1. Cultures with positive results for the oropharynx and tracheal secretions are presented in Table 3. Risk ratios for the four microbe groups show a difference in emerging fungi for oropharyngeal cultures (Table 4) in favour of the Lp treatment. There was no difference in day of appearance of the first finding of pathogens. There were no obvious trends for either type of culture or patient group (Fig. 2). Participants without any new microbial findings were 30 and 22 patients for the Lp and control groups for oropharyngeal cultures, and 31 and 30 patients for the tracheal secretion cultures, respectively.

**Table 2:** Number of patients with findings of emerging microorganisms

Microorganism		Pilot study (*n* = 50)		Second part (*n* =100)		All patients (*n* =150)	
		Lp	C	Lp	C	Lp (G+)^a^	C (G+)^a^
Airway bacteria							
	Oropharyngeal samples	1	0	1	1	2 (1)	2 (0)
	Tracheal secretions	1	0	4	1	4 (2)	1 (0)
Staphylococci							
	Oropharyngeal samples	1	0	6	6	7 (7)	4 (4)
	Tracheal secretions	1	0	7	1	8 (8)	2 (2)
Enteric bacteria							
	Oropharyngeal samples	8	13	22	16	31 (0)	29 (1)
	Tracheal secretions	4	8	15	9	19 (1)	17 (1)
Fungi							
	Oropharyngeal samples	6	8	8	14	14 (NA)	22 (NA)
	Tracheal secretions	6	3	9	11	15 (NA)	14 (NA)

Number of patients in whom new emerging bacteria or fungi were identified in oropharyngeal and tracheal secretions cultures. Two study parts and combined material separated

*Lp* patients treated with *Lactobacillus plantarum* 299, *C* control patients treated with chlorhexidine, *NA* not available

^a^Number of patients with Gram-positive bacteria (G+) in respective groups presented in parentheses

**Table 3:** Number of positive findings of bacteria and fungi species at inclusion and in subsequent samples

Species	Oropharyngeal samples				Tracheal secretions			
	Inclusion		Subsequent		Inclusion		Subsequent	
	Lp	C	Lp	C	Lp	C	Lp	C
1. *Haemophilus influenzae*	4	1	1	1	4	7	3	1
2. *Moraxella catarrhalis*	2	0	0	1	2	3	1	0
3. Beta-Streptococcus group G	1	0	1	0	0	0	0	0
4. *Streptococcus pneumoniae*	1	1	1	0	2	2	4	0
5. *Streptococcus pyogenes*	1	1	0	0	1	1	0	0
1–5. Airway bacteria	9	3	3	1	9	13	8	1
6. *Staphylococcus aureus*^a^	8	8	8	5	8	6	7	2
7. Coagulase-negative Staphylococci	0	0	2	0	0	0	0	0
8. *Aeromonas hydrophila*	0	0	0	0	0	0	0	1
9. *Chryseobacterium indologenes*	0	0	0	1	0	0	0	1
10. *Citrobacter Koseri*	0	1	0	1	0	0	0	1
11. *Citrobacter freundii*	1	0	1	0	0	0	1	0
12. *Citrobacter* sp.	0	0	1	0	0	0	1	0
13. *Escherichia coli*	4	6	2	3	4	4	1	2
14. *Enterobacter aerogenes*	3	3	1	0	0	1	2	1
15. *Enterococcus faecalis*	1	3	8	8	0	3	2	6
16. *Enterococcus faecium*	6	1	7	5	2	0	4	4
17. *Enterobacter cloacae*	2	2	2	4	2	2	1	2
18. *Hafnia alvei*	1	0	1	4	0	0	1	0
19. *Klebsiella oxytoca*	0	3	2	1	0	6	2	1
20. *Klebsiella pneumoniae*	0	3	4	1	0	3	4	1
21. *Morganella morgani*	0	0	0	1	0	0	0	0
22. *Proteus mirabilis*	0	1	3	1	1	1	0	1
23. *Proteus vulgaris*	0	0	1	1	0	1	1	1
24. *Pseudomonas aeruginosa*	0	2	2	3	2	3	0	2
25. *Pseudomonas* sp.	0	0	1	1	0	1	2	0
26. *Serratia marcescens*	2	1	1	0	0	0	3	0
27. *Serratia* sp.	0	0	0	0	1	0	0	0
28. *Stenotroph. maltophilia*	2	0	4	7	1	1	3	2
29. *Streptococcus agalactiae*	2	2	1	0	0	1	0	1
30. *Streptococcus dysgalactiae*	0	0	0	0	1	0	0	0
31. Anaerobic mixed flora	0	0	1	0	0	0	0	0
7–31. Enteric bacteria	24	28	43	42	14	27	28	27
32. *Candida albicans*	12	16	17	20	9	14	12	14
33. *Candida dubliniensis*	0	0	1	0	1	0	1	0
34. *Candida glabrata*	0	0	2	3	1	0	1	2
35. *Candida guilliermondii*	0	0	1	1	0	0	0	0
36. *Candida parapsilosis*	0	0	2	0	0	0	1	0
37. *Candida tropicalis*	0	0	1	0	0	1	1	0
38. *Aspergillus fumigatus*	1	0	0	0	1	1	0	1
32–38. Fungi	13	16	24	24	12	16	16	17

Number of positive findings of bacteria species at inclusion and in subsequent samples. Only the first sample in which a species was identified is included in the presented data. No significant differences were found between the two treatment groups

*Lp* patients treated with *Lactobacillus plantarum* 299, *C* control patients treated with chlorhexidine

^a^No methicillin-resistant *Staphylococcus aureus* was found among isolated *S. aureus* strains

**Table 4:** Risk ratio Lp/C with 95% confidence intervals

Microbe	Sample	Risk ratio (95% confidence interval)
Airway bacteria	Oropharyngeal	0.99 (0.14–6.79)
	Tracheal	4.12 (0.47–35.95)
Staphylococci	Oropharyngeal	1.81 (0.55–5.89)
	Tracheal	4.33 (0.95–19.65)
Enteric bacteria	Oropharyngeal	1.10 (0.75–1.60)
	Tracheal	1.14 (0.65–2.00)
Fungi	Oropharyngeal	0.53 (0.30–0.95)
	Tracheal	1.07 (0.56–2.05)

Risk ratios for different groups of new emerging microbes

*Lp* patients treated with *Lactobacillus plantarum* 299, *C* control patients treated with chlorhexidine

Patients with oropharyngeal cultures positive for enteric bacteria (with or without positive tracheal cultures) had a higher APACHE II score (*p* = 0.049), longer ventilator time (*p* = 0.015) and longer stay in the ICU (*p* = 0.048) compared with patients with negative cultures.

In the probiotic oral care group, Lp299 was identified in tracheal secretion samples from 31 (45%) of the patients, and enteric bacteria were also found in 15 of those subjects.

No adverse events attributable to Lp299 were registered.

VAP was identified in seven and 10 patients in the Lp group and the control group, respectively (*p* = 0.45).

## Discussion

In this multicentre study we found a similar fraction of patients with emerging potentially pathogenic bacteria for the group randomised to treatment with an oral hygiene care including the probiotic bacterium *Lactobacillus plantarum* 299 as one component, compared to that found in the group treated with standard oral care with a chlorhexidine solution. The trend towards better results for the probiotic treatment regarding new emerging enteric bacteria, found in the first part of our study, was not confirmed.

The CHX preparation (0.1%) used in our study is the standard concentration for oral care in Sweden and in several other countries. A higher concentration may be more effective in reducing pathogens but with increasing concentrations there are also more side effects reported [32]. The antiseptic CHX is active against the normal oral bacterial flora but has only a limited effect on Gram-negative bacteria [33]. CHX is commonly used for oral care in ICUs. Indeed, Koeman et al. [18] studied oral hygiene care with CHX and a combination of CHX and colistin, and noted that, compared with placebo, both of these methods reduced pathogens in oropharyngeal cultures. Furthermore, recent reviews concluded that oral care with CHX reduces the incidence of VAP but does not influence the survival rate [19, 20], which agrees with another meta-analysis showing that VAP is not associated with any change in mortality [34].

CHX reacts with lipids in the cell membrane of both bacterial and mammalian cells [35]. However, breakdown of the cell membrane leads to release of the intracellular content and thus triggers an inflammatory process, which may partly explain why a meta-analysis found increased mortality in CHX-treated patients [36, 37]. Given that aspiration (i.e. positive identification of Lp299 in the Lp group) was about the same in both groups in our study, this suggests that nearly half of patients treated with CHX as an antiseptic in oral care are at risk of harm to the lungs due to aspiration of CHX. Moreover, CHX induces allergies and has even been associated with severe anaphylactic reactions [22, 23], and these effects also occur when used orally [38]. In addition, bacterial resistance against CHX is an emerging problem [19]. Considering these drawbacks, it is clear that an alternative to CHX for oral care in ICU patients is needed.

The strain *L. plantarum* 299v reduces pathogenic bacteria overgrowth in the gut in critically ill patients [39] and adheres to the intestinal mucosa to the same extent as in healthy volunteers [26]. Therefore, we hypothesised that Lp299 would be able to outcompete oral pathogens in mechanically ventilated patients. Indeed, we found no difference in the incidence of new pathogens in the oropharynx or in tracheal samples between the two groups in our study, indicating that Lp299 might have an effect comparable to CHX to counteract oropharyngeal pathogenic colonisation. Another important aspect is that, in contrast to CHX, Lp299 has not been implicated to have any important side effects. Thus, a mixed group of ICU patients, patients with acute pancreatitis, patients with liver transplants and patients undergoing major surgery showed no adverse events related to the probiotic. In fact, in these studies the outcome was favourable with fewer infections in the probiotic groups [40–43]. In addition, Lp299v has been tested in several studies in the intensive care environment [26, 39, 40], and has been used as a nutritional additive in fruit drinks (ProViva®)for more than 25 years and has been consumed by millions of non-hospitalised individuals as well as hospitalised patients with no reported problems.

Lp299 and Lp299v have an antibiotic susceptibility profile with low MIC values for all antibiotics used in the ICU and we have demonstrated that a harvested strain of Lp299v retains its antibiotic susceptibility pattern even after exposition in the GI tract under pressure from several broad-spectrum antibiotics [44]. However, other probiotics may not have the same safety profile; in a Dutch non-ICU study in patients with pancreatitis, mortality was higher in the group that received a mixture of six probiotic strains via a naso-jejunal catheter. Nonetheless, in general, enterally administered probiotics are well tolerated and have no or only minor side effects [45].

The aim of the present study was not to show any difference between use of CHX and Lp299 with regard to development of VAP, although some investigations using other probiotic species have shown important reductions in VAP. A study by Morrow et al. [46] evaluating patients given *Lactobacillus rhamnosus* GG orally and enterally found that the incidence of VAP was 19% in the probiotic group and 40% among controls. In a recently published Chinese enteral study [5], the results were 36 and 50% respectively when *Bacillus subtilis* and *Enterococcus faecalis* were used. We have some doubt whether the results of those investigations can be generalised, because the reported incidences of VAP were strikingly high, which could question other routines applied for VAP prevention. In the present study the VAP frequency was approximately 12%, calculated for all patients.

VAP is caused by pathogenic microorganisms, directly aspirated from oropharyngeal secretions or as inhabitants present in biofilm fragments [6]. CHX reduces the incidence of VAP but is most effective against VAP caused by normal oral flora [19, 47]. Several studies and a meta-analysis have demonstrated that SOD and SDD are effective in reducing VAP in mechanically ventilated patients [9, 10, 12, 13], and may represent a suitable alternative in selected patients. In an investigation conducted by Dutch researchers [40] a regimen involving an equal mixture of Lp229 and Lp299v given enterally was compared with a SDD regimen; the results showed that the incidence of infections except UTI was similar in the two groups and there were 10 and nine cases of VAP respectively. Meta-analyses concerning the use of probiotics for VAP reduction have provided data that favour the use of probiotics [48–50].

The three presented alternative methods for reduction of VAP have different onsets. In our study the objective was to reduce pathogens in the oropharynx in order to minimise microbial load if aspiration occurs. In general, probiotics have no serious side effects [45] and thus appear to represent a seemingly harmless and ecological principle. CHX, on the other hand, has known important side effects such as development of resistance, allergic reactions and tissue toxicity, and is even associated with increased mortality in hospitalised non-ICU patients [51], and SDD or SOD methods carry the risk of development of resistant bacteria. In recent years, research on what is called the resistome has presented clear evidence that SOD and SDD do increase the occurrence of resistant species in the GI tract [14–16]. Colistin is a component in most SDD and SOD regimens. It should be noted that the frequency of colistin-resistant bacteria is increasing. Since colistin in many cases is in the last line of defence, it ought to be used in selected cases and to fight infections [17, 52, 53].

There are many limitations to our study. First, the study was performed in two steps and routines may have changed over time. Second, we did not include enteral administration of Lp299, but such an approach would have invalidated a comparison between CHX and Lp299. Third, the study was not powered for non-inferiority and the number of patients was limited, hence we cannot exclude the possibility that a minor difference exists between the treatment policies.

The clinical implication of our study is that Lp299 could be considered as an alternative for oral hygiene care in mechanically ventilated patients.

## Conclusion

Emerging potentially pathogenic bacteria were found in a similar fraction in the group of patients treated with oral hygiene care including the probiotic bacterium *Lactobacillus plantarum* 299 as one component, compared to that found in the group treated with standard oral care with a chlorhexidine solution. We did not examine any other potential use of Lp299 or any other probiotic, and therefore we cannot conclude whether this probiotic strain is the ideal choice to limit colonisation of pathogens. Clearly, more high comprehensive investigations are needed to provide sufficient evidence to indicate which probiotic strains or combinations thereof may be the most suitable and for what purpose.

## Key messages

- Lp299 used for oral care in intubated, mechanically ventilated, critically ill patients is as effective as CHX in inhibiting the incidence of emerging potentially pathogenic bacteria in the oropharynx.
- No adverse effects was observed when the oral care procedure was performed using the probiotic bacterium Lp299.
- Probiotics may be a suitable alternative to other procedures aimed at reducing the burden of pathogenic microorganisms in the mouth and the oropharynx, and thereby may also lower the risk of developing VAP. When using probiotics, the risk for development of resistant bacteria is essentially negligible.

## Abbreviations

APACHE: Acute Pathophysiology and Chronic Health Evaluation
CHX: Chlorhexidine
ETT: Endotracheal tube
GI: Gastrointestinal
ICU: Intensive care unit
LIS: Lung Injury Score
LOS: Length of stay
Lp299: *Lactobacillus plantarum* 299
SDD: Selective digestive decontamination
SOD: Selective oropharyngeal decontamination
UTI: Urinary tract infection
WBC: White blood cell
